# Transcription Factor Occupancy Can Mediate Active Turnover of DNA Methylation at Regulatory Regions

**DOI:** 10.1371/journal.pgen.1003994

**Published:** 2013-12-19

**Authors:** Angelika Feldmann, Robert Ivanek, Rabih Murr, Dimos Gaidatzis, Lukas Burger, Dirk Schübeler

**Affiliations:** 1 Friedrich Miescher Institute for Biomedical Research, Basel, Switzerland; 2 Swiss Institute of Bioinformatics, Basel, Switzerland; 3 Faculty of Science, University of Basel, Basel, Switzerland; Stanford University School of Medicine, United States of America

## Abstract

Distal regulatory elements, including enhancers, play a critical role in regulating gene activity. Transcription factor binding to these elements correlates with Low Methylated Regions (LMRs) in a process that is poorly understood. Here we ask whether and how actual occupancy of DNA-binding factors is linked to DNA methylation at the level of individual molecules. Using CTCF as an example, we observe that frequency of binding correlates with the likelihood of a demethylated state and sites of low occupancy display heterogeneous DNA methylation within the CTCF motif. In line with a dynamic model of binding and DNA methylation turnover, we find that 5-hydroxymethylcytosine (5hmC), formed as an intermediate state of active demethylation, is enriched at LMRs in stem and somatic cells. Moreover, a significant fraction of changes in 5hmC during differentiation occurs at these regions, suggesting that transcription factor activity could be a key driver for active demethylation. Since deletion of CTCF is lethal for embryonic stem cells, we used genetic deletion of REST as another DNA-binding factor implicated in LMR formation to test this hypothesis. The absence of REST leads to a decrease of hydroxymethylation and a concomitant increase of DNA methylation at its binding sites. These data support a model where DNA-binding factors can mediate turnover of DNA methylation as an integral part of maintenance and reprogramming of regulatory regions.

## Author Summary

Cell identity is determined by differential gene expression, which in turn is controlled by the combined activity of proximal and distal regulatory elements such as enhancers. DNA within active enhancer elements is marked by a hypomethylated state as a result of transcription factor (TF) binding. Here, using CTCF as an example for a DNA-binding factor, we explore the relationship between binding and DNA methylation at the level of single molecules by enriching for CTCF occupied DNA. To our surprise, methylation at molecules which are bound by CTCF does not differ from the average methylation levels at the binding sites defined by whole-genome bisulfite sequencing. We find that binding strength inversely correlates with DNA methylation within the CTCF motif with heterogenic methylation levels at low occupancy sites, suggesting that CTCF can bind to molecules with different methylation states. Moreover, we observed enrichment of 5-hydroxymethylcytosines at constitutive and cell-type specific TF binding sites indicative of an active demethylation process. To test the requirement of TF binding for the observed hydroxymethylation, and as CTCF deletion is incompatible with the survival of embryonic stem cells, we made use of cells in which REST – a factor which was previously shown to be involved in LMR formation - was genetically deleted. This deletion leads to loss of hydroxymethylation at its binding sites, suggesting that binding is necessary for turnover. Our data support a model in which TF occupancy mediates a continuous turnover of DNA methylation during maintenance and formation of active regulatory regions.

## Introduction

Correct spatial and temporal regulation of genes depends on distal regulatory elements. Reprogramming the activity of these elements is thus central for successful cellular specialization [1], [2]. Active distal regulatory elements are characterized by an open chromatin structure, corresponding to DNaseI hypersensitive sites, specific histone variants and histone modifications [3], [4]. These modifications are thought to regulate the accessibility of the regulatory sequence and thus facilitate transcription factor (TF) binding [5].

Distal regulatory regions that reside outside of CpG islands are further unique, as they show reduced levels of DNA methylation when active [6]–[8]. Importantly, this feature is consistent between cell types so that it can be implemented to identify cell-type specific active regulatory elements as Low Methylated Regions (LMR) [6], [7], [9]–[11]. Although reduced, DNA methylation at LMRs is maintained at a residual level. This reflects heterogeneity within the population of sequenced DNA molecules, given that DNA methylation is binary for any particular cytosine. Functional experiments suggested that reduced methylation at LMRs critically depends on binding of transcription factors [7], but their role in creating methylation heterogeneity and whether this occurs via a passive and/or an active demethylation remains to be identified.

Several lines of evidence further link DNA demethylation to enhancer activity. Demethylation occurs at glucocorticoid receptor binding sites [8] and 5-hydroxymethylcytosine (5hmC), an intermediate of active demethylation via oxidation of 5-methylcytosines (5mC) by TET proteins [12]–[16], is present at active enhancers in embryonic stem (ES) cells as well as during neuronal and adipocyte differentiation [7], [17]–[21]. Importantly, 5hmC can readily be detected in various cell types and thus utilized to locate regions of active DNA demethylation [22], [23].

Here we addressed, whether heterogeneous methylation at LMRs reflects differential occupancy by transcription factors at individual molecules, using the DNA binding factor CTCF as an example. We show that CTCF-bound molecules display similar methylation levels as those observed in the entire cell population at CTCF binding sites. Moreover, for cytosines located within the CTCF motif, we find that binding affinity correlates with the likelihood of being unmethylated, so that CTCF is able to bind any methylation state within low occupancy sites. On the other hand, we find that high levels of hydroxymethylation coincide with the observed low methylation at LMRs, in a process that accounts for up to 20% of the genome-wide dynamics of 5hmC during neuronal differentiation of ES cells. Moreover, the presence of hydroxymethylation depends, at least partially, on TF binding, since genetic deletion of RE1-silencing transcription factor (REST) results in reduced hydroxymethylation at bound LMRs. Our results support a model where TF binding can occur at methylated regions and induce methylation turnover within active regulatory elements.

## Results

### Relation between CTCF occupancy and methylation states at CpG poor regions

**Figure 1:**
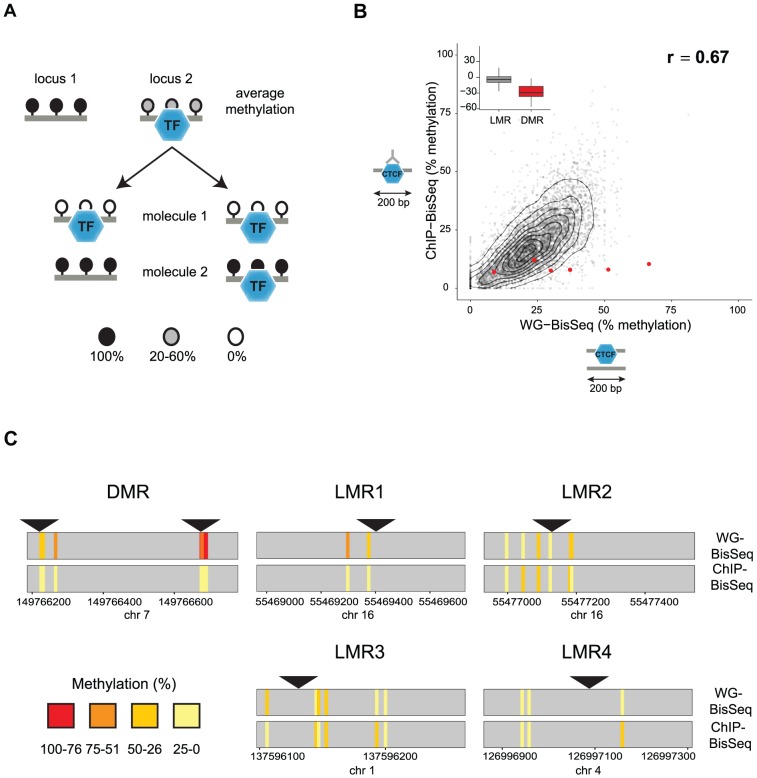
Relation between CTCF occupancy and methylation states in CpG poor regions. (A) LMRs are bound by transcription factors (TF) and have intermediate average methylation levels. There are two possible scenarios how TF binding and DNA methylation at CpG poor regions could be linked. In a static situation (left), TF binding would be linked to the unmethylated state of the bound molecule, whereas unbound molecules are fully methylated as previously shown for imprinted CpG islands. In an unlinked model (right), TF binding is independent of the DNA methylation state, therefore bound molecules display the same variation of methylation levels as the entire population. (B) To distinguish these scenarios we enrich for bound molecules by ChIP and determine their methylation by bisulfite sequencing (ChIP-BisSeq). This results in a high correlation of methylation levels between ChIP-BisSeq (y-axis) and whole genome bisulfite sequencing (WG-BisSeq, x-axis). Each point represents average methylation over a 200 bp region. Shown are only regions centered at a bound CTCF motif which overlaps with an LMR and for which all considered cytosines have a minimal coverage of 10× in both, WG-BisSeq and ChIP-BisSeq. Red points represent average for 200 bp windows centered on CTCF motifs located within DMRs. Boxplots show mean deviation of methylation levels in ChIP-BisSeq from those in WG-BisSeq at LMRs and DMRs in percent methylation. (C) Examples of single cytosine methylation levels in WG-BisSeq (top bars) and ChIP-BisSeq (bottom bars). For LMRs a whole segment is shown. Each bar represents a cytosine. Methylation is shown in a color code (red: high, yellow: low). Position of CTCF motifs is indicated by black triangles. Only cytosines with at least 10× coverage in both, WG-BisSeq and ChIP-BisSeq, are shown.

Apart from CpG islands, mammalian genomes are mostly methylated. Notable exceptions are LMRs, CpG poor regions that display an average methylation level of 30% as measured by bisulfite sequencing (BisSeq). This reduced methylation marks active distal regulatory regions as it coincides with DNaseI hypersensitivity and enhancer-characteristic histone modifications [7]. We previously showed that, in the case of REST and CTCF, binding of trans-acting factors to DNA is required for LMR formation, yet it remains unclear whether and how this binding is related to the observed variation of DNA methylation between sequenced molecules [7]. Assuming a static model, unmethylated DNA would be limited to those molecules that are occupied by a TF, which in turn predicts that methylated molecules are not occupied, as has been established for imprinted CpG islands (Figure 1A, left) [24], [25]. Alternatively, TFs could occupy all variations of methylation levels within LMRs (Figure 1A, right).

To test the first scenario, we performed Chromatin-IP (ChIP) in ES cells against the DNA binding factor CTCF and conducted bisulfite sequencing of the immunoprecipitated CTCF-bound DNA (ChIP-BisSeq) (Figure 1B) [26], [27]. Importantly, the CTCF-ChIP enrichments recovered in our ChIP-BisSeq samples highly correlate with published ChIP enrichments [7] (r = 0.91 and 0.90 for replicate1 and replicate2, respectively) as well as between the replicate experiments (r = 0.91) (Figure S1A). Equally important, methylation for single cytosines correlates between the two replicates (r = 0.8, Figure S1B).

Only those CpGs, which show intermediate levels of methylation in BisSeq, can be informative to address our hypothesis. Therefore we first focused on CTCF sites located within LMRs. In this context it should be mentioned that the mean methylation of 30% observed at an LMR represents an average of individual cytosines within this LMR that can vary widely in their methylation percentage ([7] and data not shown). To ask if this heterogeneity is reduced at the occupied molecules, we compared methylation levels between the CTCF-bound fraction and the total population of cells. We first analyzed CpGs residing in sites of known allelic variation in CTCF binding, corresponding to DMRs, where we indeed only recover the unmethylated alleles in the ChIP-BisSeq assay (Figure 1B–C). This agrees with a recent report and confirms that our ChIP-BisSeq provides correct methylation status of bound molecules [25], [26].

Next we asked if methylation patterns at CTCF-bound LMRs differ between exclusively bound molecules and the total population of DNA molecules. We analyzed average methylation levels for 200 bp regions centered at a CTCF motif only for those motifs which (1) overlap with LMRs, (2) are bound by CTCF as determined by ChIP enrichments and (3) for which all considered cytosines are covered at least 10 times in both ChIP-BisSeq and whole-genome (WG-) BisSeq. It is important to mention here, that while our ChIP does not allow for calling high resolution peaks such as those determined by other methods like ChIP-exo [28], our analysis pipeline is able to correctly identify high confidence bound sites as it requires CTCF motif in the center of the analyzed region in addition to high ChIP enrichment. This revealed a positive correlation with an equal spread of the data over the entire range (r = 0.67), arguing that LMRs do not display global differences in methylation levels at CTCF binding sites between the fraction of molecules bound by CTCF and those representing the total population of molecules in cells (Figure 1B). This finding is illustrated at individual loci (Figure 1C) and extends to CTCF binding sites outside of LMRs (Figure S1C).

We notice however that while entire LMRs do not display reduced methylation in the actually bound fraction of molecules, some individual cytosines in the vicinity of the CTCF motif do so (for example LMR1 in Figure 1C). To determine whether reduced methylation in CpGs close to the CTCF motif is a global phenomenon, we correlated changes in methylation between ChIP-BisSeq and WG-BisSeq with the distance to the nearest CTCF motif for individual cytosines with a minimal coverage of 10 fold in WG-BisSeq and ChIP-BisSeq in CTCF-bound Low Methylated Regions. This analysis revealed no correlation, suggesting that CTCF binding does not affect the methylation of proximal cytosines more than it does for the distal ones (Figure S1D). Therefore, the heterogeneity of occupancy by CTCF cannot explain the observed heterogeneity of methylation within LMRs, even though these can form upon CTCF binding and thus, at least in part, are CTCF dependent [7].

**Figure 2:**
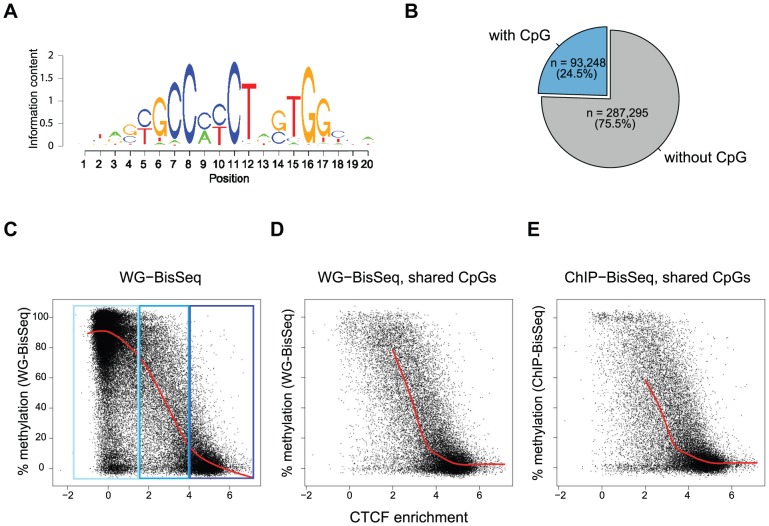
Relationship between binding strength and DNA methylation within the CTCF motif. (A) CTCF consensus motif used in this study [7]. (B) Percent of predicted CTCF sites containing a CpG within the motif. Exclusively these CpGs are shown in the plots (C–E). (C–E) Each point represents one individual CpG within a CTCF motif. (C) Correlation of methylation and CTCF enrichment identifies three classes of CTCF sites: unbound (light-blue), strongly bound and unmethylated (dark-blue), weakly bound with intermediate levels of methylation (blue). The red line represents a running mean measurement of methylation. (D) Same as C, but only showing cytosines covered in both WG-BisSeq and CTCF ChIP-BisSeq. (E) Same as D but only showing methylation levels derived from CTCF ChIP-BisSeq. In each case bound molecules show the same pattern as the entire population. Only cytosines residing within the CTCF binding motif and with a minimal coverage of 10× are shown. In order to prevent over-plotting the points were jittered with a standard deviation of 2%.

To further test the relationship between occupancy and methylation state, we next focused our analysis exclusively on CpGs that reside within a CTCF motif. We and others have previously shown that methylation around occupied CTCF sites is the lowest at the actual binding motif and increases outwards [7], [21]. Notably, 57% of all occupied sites by CTCF do not contain a CpG within the binding motif, yet display the same methylation pattern around the site (Figure 1B, Figure S1 and data not shown). Out of all predicted CTCF binding sites, 24.5% contain at least one CpG (Figure 2A–B). For all these sites, we related the strength of binding by CTCF as measured by ChIP enrichment to the methylation state of single CpGs within the motif. This reveals that strongly and weakly bound sites indeed differ in their methylation (Figure 2C). CpGs within highly occupied sites tend to be completely unmethylated, while methylation shifts towards intermediate levels with decreasing binding affinity. This links frequency of occupancy to methylation levels within the CTCF motif.

Again we can ask if heterogeneous methylation at weakly bound sites reflects actual occupancy at the level of individual molecules by analyzing their methylation in the bound fraction that was enriched by CTCF-ChIP. Also at these selected CpGs the methylation of exclusively occupied molecules is similar to the methylation of the total population (Figure 2D–E). Importantly, this relationship between the methylation state and CTCF binding is not dependent on the position of the analyzed CpG, as illustrated by the analysis of CpGs positioned exclusively at position 5–6 of the consensus motif (Figure S2).

Together, our data suggest that actual factor occupancy at the level of single molecules does not explain the observed DNA methylation heterogeneity adjacent to CTCF sites within LMRs or at the motif itself throughout the genome. This argues against a scenario of static methylation at CpG poor regions (Figure 1A, left), where DNA in a fraction of cells is bound by a TF and unmethylated, while other molecules are never occupied and remain methylated. Alternative scenarios could involve binding of a TF independently of methylation states, which in turn could trigger active demethylation (Figure 1A, right).

### Hydroxymethylation marks LMRs in a cell-type specific and transcription factor binding dependent fashion

**Figure 3:**
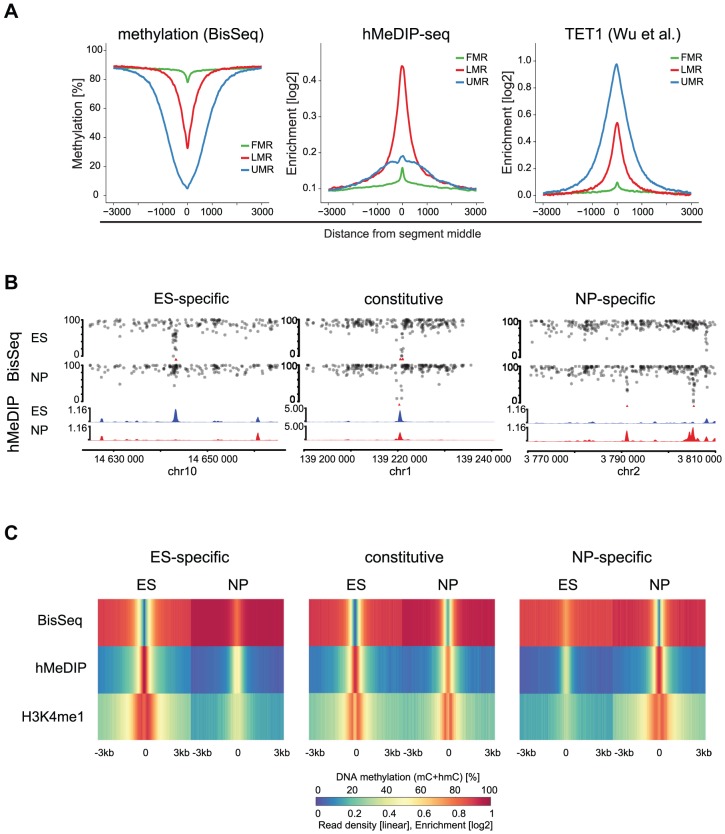
5hmC marks LMRs in a cell-type specific fashion. (A) Average profiles for methylation (WG-BisSeq), 5hmC (hMeDIP-seq) and TET1 occupancy at Fully Methylated, Unmethylated and Low Methylated Regions (FMRs, UMRs and LMRs, respectively) in ES cells. (B) DNA methylation (upper tracks) and enrichment of 5hmC (lower tracks) in ES cells and NP of representative ES-specific, constitutive and NP-specific LMRs. (C) Average profiles representing methylation (WG-BisSeq), hMeDIP-seq and H3K4me1 ChIP-Seq in ES cells and NP ±3 kb around the segment middle.

To ask if LMRs are indeed sites of active DNA methylation turnover, we determined the presence of 5hmC, the intermediate of TET mediated oxidation. Notably, bisulfite does not convert 5hmC and thus a fraction of the residual unconverted cytosines at LMRs could represent hydroxymethylcytosines [29], [30]. We enriched for this modification by performing hydroxymethylcytosine DNA-immunoprecipitation (hMeDIP) followed by high throughput sequencing (hMeDIP-seq) in stem cells [31], [32]. Analysis of the 5hmC profiles revealed its enrichment at LMRs of ES cells in line with other reports that suggested its presence at stem cell enhancers (Figure 3A) [19], [21]. Analysis of an existing map of genomic binding sites further reveals that also TET1, an enzyme that mediates oxidation to 5hmC, is strongly enriched at LMRs in ES cells (Figure 3A) [33].

To address, whether the presence of 5hmC at LMRs is limited to stem cells or conserved in committed cells, we performed hMeDIP-Seq in neuronal progenitors (NP), derived through controlled differentiation of ES cells [34]. We previously showed in the same differentiation system that a large set of LMRs is cell-type specific, reflecting the extensive reprogramming of distal regulatory regions during somatic differentiation [7]. The resulting genomic 5hmC profiles reveal its enrichment at LMRs also in NP (Figure 3B–C). LMRs that are constitutive in both cell types show constitutive hydroxymethylation, suggesting that oxidation of 5-methylcytosine at LMRs also occurs in somatic cells (Figure 3B–C). ES-specific LMRs gain methylation and concomitantly lose hydroxymethylation in NP, suggesting that the state of reduced methylation and the presence of 5hmC coincide at active regulatory elements (Figure 3B–C, Figure S2). Similarly, NP-specific LMRs show a decrease in methylation and gain of hydroxymethylation along differentiation (Figure 3B–C, Figure S3). Notably, these NP-specific LMRs are enriched for neuron-specific TF binding sites, further confirming the link between TF binding at CpG poor regions and the presence of 5hmC [7]. The observed reciprocal behavior between loss of 5mC and gain of 5hmC is a general feature, as a genome-wide anti-correlation between changes in hMeDIP-Seq and WG-BisSeq (r = −0.58) as well as between changes in hMeDIP-Seq and MeDIP-Seq (r = −0.30, Figure S3) exists at LMRs.

**Figure 4:**
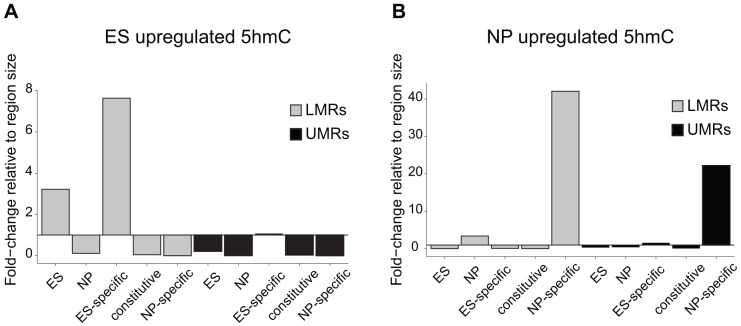
5hmC dynamics during differentiation occurs preferentially at LMRs. (A–B) Shown is the relative frequency of changes in 5hmC at LMRs and UMRs normalized for genome coverage at the ES (A) and NP state (B). The y-axis shows observed linear fold enrichment relative to expected enrichments (see Materials and Methods). Note that 5hmC is changing preferentially at cell-type specific LMRs.

To determine, if the observed turnover is selective for LMRs, we quantified 5hmC enrichments by hMeDIP-Seq throughout the genome and calculated the differences between ES cells and NP in order to identify genomic regions that show changes in the level of 5hmC. This revealed that cell-type specific enrichments for 5hmC show a large overlap with cell-type specific LMRs. This selectivity is further evident when calculating the occurrence in relation to genomic coverage (Figure 4). In this analysis, ES-specific LMRs are eightfold overrepresented in genomic regions that show enrichment for 5hmC in ES cells and the selectivity is even higher in NP, where NP-specific LMRs are more than 40-fold overrepresented.

**Figure 5:**
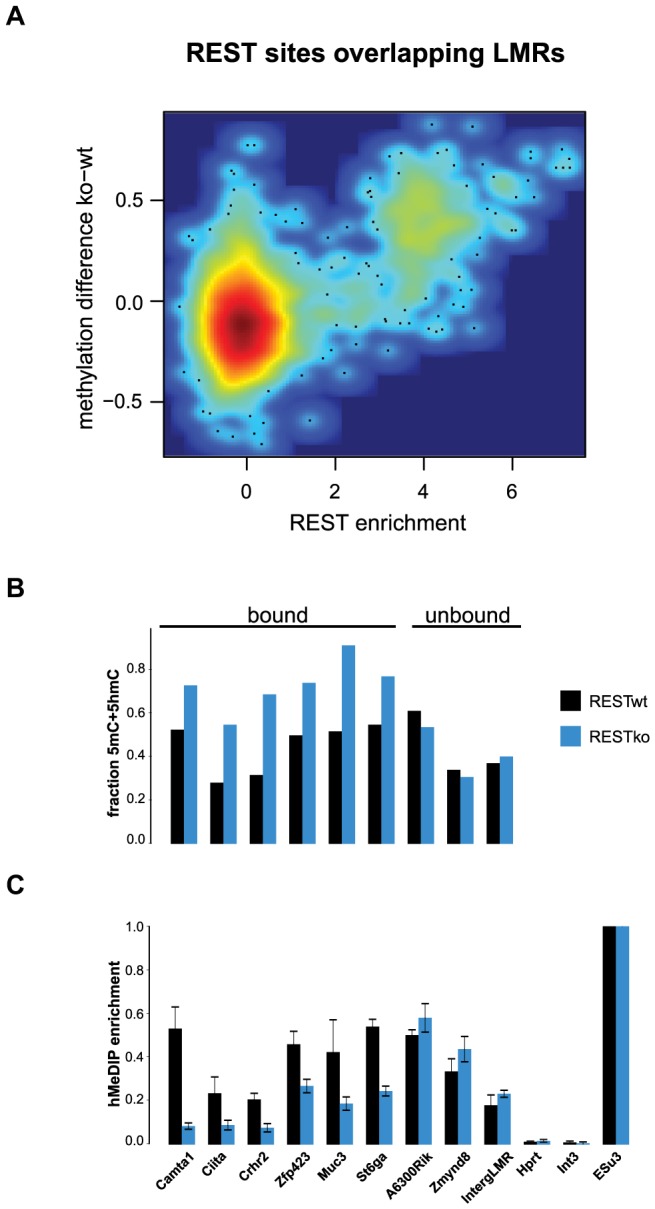
5hmC enrichment at REST-bound LMRs is partially dependent on the presence of REST. (A) Relative methylation changes between REST wildtype and REST knockout ES cells are correlated to REST ChIP enrichment. Methylation was determined 200 bp around the REST motif at all REST sites overlapping with LMRs. The point density is colour-coded (red: high, blue: low point density). Methylation determined by BisSeq (B) and hMeDIP qPCR enrichments (C) at REST motif containing LMRs bound and not bound by REST in wildtype (wt, dark blue) and REST knockout (ko, blue) ES cells. Error bars in (C) represent standard deviation in three replicate experiments normalized to a positive control.

This strong correlation suggests that transcription factors are required to induce hydroxymethylation. Indeed, 5hmC is more enriched at bound than at unbound CTCF motifs (Figure S4). To directly test whether increased 5hmC enrichment is a consequence of TF binding, we wanted to use a loss of function approach. Absence of CTCF, notably in ES cells, is cellular lethal [35]–[38], which precludes monitoring changes in methylation in cells that lack CTCF but otherwise are phenotypically normal. Effective depletion of CTCF would however be required in order to directly test its requirement in trans, since conserved binding sites remain occupied upon knockdown of CTCF [39]. As CTCF deletion is incompatible with survival of ES cells, we made use of a phenotypically normal ES cell line in which the *Rest* gene, coding for a different TF that is enriched within LMRs, had been genetically deleted. More specifically, we determined the level of hydroxymethylation at REST-bound LMRs. These regions become fully methylated in the absence of REST as measured by bisulfite sequencing, which is not discriminating between 5mC and 5hmC (Figure 5A–B). When measuring hydroxymethylation specifically by hMeDIP (see Table S1 for primers) we find that 5hmC levels are significantly reduced at these binding sites in REST knockout ES cells (Figure 5C). This indicates that factor activity in trans is required for increased hydroxymethylation at LMRs within a given cell type.

These observations are compatible with a scenario in which reduced DNA methylation at regulatory regions entails the presence of active DNA methylation turnover in both stem and differentiated cells.

## Discussion

Using CTCF as example, this study provides further evidence that maintenance and reprogramming of correct DNA methylation levels at distal regulatory regions can entail active turnover as a function of transcription factor binding. We show that the loss of methylation at these regions during cellular differentiation involves a reciprocal gain of 5hmC and vice versa. This process occurs preferentially at LMRs and we demonstrate that it accounts for up to 20% of all observed changes in 5hmC during differentiation. These findings are compatible with previous reports of dynamic hydroxymethylation [18], [40]. Importantly, this association is not limited to stem cells, even though these have been suggested to display higher global levels of 5hmC than differentiated cells [16]. We also show that this phenomenon can go beyond correlation, since genetic deletion of the TF REST results in reduced hydroxymethylation at its binding sites already in stem cells. Our results obtained from CTCF and REST mechanistically link binding of TF at regulatory regions with active demethylation. However, in light of the estimated 1400 different TFs encoded in mammalian genomes, it would be premature to generalize these findings.

The fact that CTCF can occupy different methylation states in CpG poor regions together with the presence of both 5hmC and TET1 at these sites is compatible with a scenario, where TF binding triggers an active demethylation process. In case of CTCF it is evident that the binding strength determined by ChIP relates directly to the level of demethylation within the binding motif. The frequency of binding correlates with the likelihood of a demethylated state for a cytosine within the binding site. Assuming that this relation extends to factors other than CTCF adds yet another dimension to whole-genome basepair methylomes by providing not only information about the activity of regulatory regions, but also about the strength of binding of trans-acting factors. It is important to note however that both CTCF and REST are rather special in regards to the large size of their sequence motifs (20 and 21 bp, respectively), which further limits the ability to generalize our observations. Clearly, a more comprehensive approach is needed to address the effect of additional DNA-binding factors on DNA methylation.

While the actual mode of demethylation remains to be determined, it seems possible that DNA binding factors recruit TET proteins, which in turn mediate oxidation to 5hmC [41]. However, in light of the generality of the link between LMR formation and 5hmC, this would require a large number of TFs to share such recruitment ability. Alternatively, recruitment might be mediated by general cofactors that are frequently observed at distal regulatory regions such as p300 or by pioneer factors [3], [42]. A further scenario could be that a specific nucleosome or DNA organization results from binding of a TF, which in turn triggers TET recruitment [43].

At this point we can only speculate if 5hmC presence at regulatory regions solely reflects active turnover [21], [44]–[48] and how much an active process contributes to the low levels of methylation observed. Moreover, it remains to be shown if presence of hydroxymethylation is actually involved in enhancer regulation. This would require specific readers of this DNA modification. Indeed, several proteins have been suggested to bind 5hmC, including the MBD domain proteins MeCP2 [49] and MBD3 [50]. Our recent functional mapping, however, suggested that genomic binding sites of MBD3 are independent of the presence of hydroxymethylation [51] in agreement with *in vitro* binding [52], making this scenario less likely. In addition, other putative readers of 5hmC were suggested in a proteomics screen, yet only few appear to be selective for 5hmC *in vitro* [52]. Conversely, two recent studies report the accumulation of TET-mediated 5hmC oxidation products 5-formylcytosine and 5-carboxylcytosine at proximal and distal regulatory elements in the absence of TDG [46], [47], arguing for the appearance of an active turnover at LMRs. It remains to be determined, whether DNA binding factors, such as CTCF and REST used here, are able to bind to hydroxymethylated regions. While strong CTCF binding sites are devoid of methylation and hydroxymethylation, it is possible that CTCF is able to bind to 5mC as well as to 5hmC at low occupancy sites.

Our findings argue that LMRs do not result solely from a passive loss of methylation during replication, which is in line with the observation that LMRs can be detected in methylomes from non-dividing cells [9] and with recently reported presence of 5-formylcytosine and 5-carboxylcytosine at these elements [46], [47]. At this point we lack experimental evidence for the relevance of reduced methylation for the function of distal regulatory regions. It is conceivable, but remains to be shown, that reduced methylation induced by pioneering TFs would enhance binding of other TFs, which are sensitive to DNA methylation even in CpG poor regions [53], [54]. Alternatively, but not mutually exclusive, reduced methylation could mediate a chromatin state that functions as a general attractor for DNA binding factors and thus would stabilize the on-state [55].

## Materials and Methods

### ES cell culture and differentiation

159-2 ES cells were cultured and differentiated as previously described [7], [34].

### CTCF ChIP-bisulfite sequencing

Chromatin immunoprecipitation (ChIP) assay for CTCF was performed according to the Upstate protocol using the antibody anti-CTCF (SantaCruz #15914). 100 ng of immunoprecipitated DNA were used for subsequent library preparation. DNA fragments were end repaired by incubation at 20°C for 30 minutes with 400 µM dNTP, 3 units of T4 DNA polymerase (NEB #M0203S), 5 units of DNA Polymerase I Lg. Frag. (Klenow) (NEB #M0210S), 10 units of T4 PNK (NEB #M0201S), 1× T4 DNA ligase buffer containing 10 mM ATP (NEB), followed by column purification using QIAquick PCR Purification Kit (QIAGEN #28106). 3′ ends of DNA fragments were adenylated by incubation at 37°C for 30 minutes with 200 µM dATP, 1×NEB Buffer 2, 5 units Klenow Fragment (3′→5′ exo–) (NEB # M0212L), followed by column purification using MinElute PCR Purification Kit (QIAGEN # 28006). Adapter for single end sequencing were reproduced based on Illumina adapter sequences. Annealed adapters were ligated to the DNA fragments by incubation at room temperature for 15 min in the following mix: 400 nM of annealed adapters, 1× NEB Quick ligase buffer, 2.000 units of T4 Quick ligase (NEB #M2200S), followed by column purification using MinElute PCR Purification Kit. 200 ng of Drosophila DNA (Kc cells) were then added as a carrier. Adapter-ligated DNA of 150–400 bp was selected from 2% agarose gel electrophoresis and purified using MinElute Gel Extraction Kit (QIAGEN #28606). BSA (final concentration 0.5 µg/µl) was added to gel-purified DNA and the mix was then treated with sodium bisulfite using the Imprint DNA Modification Kit (Sigma-Aldrich) as per manufacturer's instructions. DNA was enriched using 18 cycles of PCR with the following reaction composition: 2.5 U of uracil-insensitive *PfuTurboCx* Hotstart DNA polymerase (Stratagene), 5 µl 10× *PfuTurbo* reaction buffer, 25 µM dNTPs, 0.5 µM of Single End Illumina PCR primers (1.1 and 2.1). The thermocycling parameters were: 95°C 2 min, 98°C 30 sec, then 18 cycles of 98°C 15 sec, 65°C 30 sec and 72°C 3 min, ending with one 72°C 5 min step, followed by column purification using the MinElute PCR Purification Kit. DNA was then run on 2% agarose gel to separate the library from adapter dimers and purified using the MinElute Gel Extraction Kit. Quality of the libraries and template size distribution were checked on an Agilent 2100 Bioanalyzer (Agilent Technologies).

### RESTko bisulfite sequencing

Library for the shotgun whole-genome BisSeq for RESTko cells was prepared as previously described [7] and sequenced using one lane of Illumina HiSeq 2000.

### hMeDIP and MeDIP sequencing library preparation

Genomic DNA was fragmented to 200–1000 bp fragments with a Bioruptor (Diagenode, Sparta, NJ). The protocol for the library preparation was adapted from Illumina Genomic DNA Sample Preparation Guide. Briefly, 7 to 10 µg of fragmented DNA were end repaired and their 3′ ends adenylated. Genomic single end or paired end adapters were annealed. (h)MeDIP was performed as previously described [56] using 4 ug of adapter-ligated DNA and 4 µl of a 1∶10 dilution of rabbit polyclonal anti-hmC antibody (Active Motif #39770) for hMeDIP or 10 µl of mouse monoclonal 5mC antibody (Eurogentec #BI-MECY-1000) for 2 hrs, followed by addition of 40 µl of Protein A Dynabeads (Invitrogen, #100.02D, hMeDIP) or Dynabeads M-280 Sheep anti-mouse IgG (Dynal Biotech #112.01) added for another 2 hrs. Immunoprecipitated DNA was amplified by 18 cycles of PCR following the Illumina Genomic DNA Sample Preparation Guide and purified using the MinElute PCR purification kit. Fragments of 250–300 bp (for single end sequencing) or 400–450 bp (for paired end sequencing) were size-selected from 2% agarose gel and purified using the MinElute Gel Extraction Kit. Quality of the libraries and template size distribution were checked on an Agilent 2100 Bioanalyzer (Agilent Technologies).

### High-throughput sequencing

(h)MeDIP-seq and ChIP-BisSeq were sequenced using the Illumina HiSeq 2000 as per manufacturer's instructions.

### Analysis of sequencing data

The hMeDIP-seq data were analyzed similarly to ChIP-Seq data in Stadler et al. Briefly, the July 2007 M. musculus genome assembly (NCBI37/mm9) provided by NCBI (http://www.ncbi.nlm.nih.gov/genome/guide/mouse/) and the Mouse Genome Sequencing Consortium (http://www.sanger.ac.uk/Projects/M_musculus/) was used as a basis for all analyses. For reads from hMeDIP-seq experiments, alignments to the mouse genome were performed by the software bowtie (version 0.9.9.1) [57] with parameters -v 2 -a -m 100, tracking up to 100 best alignment positions per query and allowing at most two mismatches. Each alignment was weighted by the inverse of the number of hits. All quantifications were based on weighted alignments. Alignments were shifted by 60 bases (estimated fragment length was 120 bp).

In order to identify regions with different signal in hMeDIP-seq between ES and NP, the mouse genome was partitioned into 1 kb sized windows with an overlap of 500 bp. For each window we calculated log2 fold change between NP and ES using in the following way: log2(FC) = log2((n_NP/N_NP *min(N_ES,N_NP)+p)/(n_ES/N_ES *min(N_ES,N_NP)+p)), where n_ES and n_NP are the summed weights of overlapping ES and NP read alignments, respectively. N_ES and N_NP are the total number of aligned reads in ES and NP samples and p is a pseudocount constant (p = 8) used to regularize enrichments based on low counts that would otherwise be dominated by sampling noise. Windows with log2(FC) bigger than 3 or smaller than −3 in both biological replicates were merged into regions showing the gain and loss of signal in NP, respectively. These regions were used to calculate the enrichment in segment types (constitutive, ES- or NP-specific LMRs, UMRs). Enrichments were calculated as the ratio of observed over expected number of bases of each region class (gain of signal in NP, loss of signal in NP) in a segment type (e.g. ES-specific LMR etc.), where the observed number is the number of bases in regions of a given class that overlap a segment and the expected number is the fraction of genomic bases in that segment type, multiplied with the total number of bases in all regions of that class.

Analysis of ChIP-Seq and bisulfite (ChIP-BisSeq) data, ChIP enrichment calculation and identification of CTCF binding sites were performed as previously described (Stadler et al. 2011). The data from the two CTCF ChIP-BisSeq replicates were pooled for the analysis. Analysis of REST ChIP-Seq data and genome-wide prediction of REST motifs was performed analogously to CTCF. In the case of REST, the inferred weight matrix was extended to allow for a variable linker (0–11 nts in length) after position 9.

### Datasets used in this study

Datasets generated for this study, ChIP-BisSeq, hMeDIP-seq, MeDIP-seq and RESTko methylome have been submitted to GEO and are available under the accession number GSE39739. Data for CTCF ChIP-Seq and WG-BisSeq was downloaded from GEO: GSE30206 [7], data for REST ChIP-Seq were downloaded from GSE27148 [58]. Tet1 ChIP-Seq data was downloaded from GEO: GSE26833 [33].

## Supporting Information

Figure S1 Genome-wide relation between transcription factor occupancy and methylation states. (A) Correlation of ChIP enrichments between CTCF ChIP-Seq (Stadler et al., Nature 2011) and the two CTCF ChIP-BisSeq replicates used in this study. (B) Correlation of methylation levels at individual CpGs between two CTCF ChIP-BisSeq replicates. Selected cytosines have a minimal coverage of 10 in both replicates. (C) Correlation of average methylation levels at regions 200 bp around all predicted CTCF sites between WG-BisSeq and a pool of both CTCF ChIP-BisSeq replicates. Selected regions have a minimal coverage of 10 in all cytosines used for the calculation of methylation levels in both WG-BisSeq and ChIP-BisSeq. (D) For individual cytosines within LMRs the methylation difference between ChIP-BisSeq and WG-BisSeq is correlated with the distance to the nearest CTCF motif center.
(PDF)

Figure S2 Relationship between binding strength and DNA methylation within the CTCF motif. (A) CTCF consensus motif used in this study. Here only cytosines are analyzed which are at position 5–6 of the motif. Out of all predicted sites containing a CpG within the motif (24.5% of all predicted sites) 42.2% have a CpG at this position. (B–D) Each point represents one individual CpG at position 5–6 of the PWM. (B) Correlation of methylation and CTCF enrichment identifies three classes of CTCF sites: unbound (light-blue), strongly bound and unmethylated (dark-blue), weakly bound with intermediate levels of methylation (blue). The red line represents a running mean measurement of methylation. (C) Same as B, but only showing cytosines covered in both WG-BisSeq and CTCF ChIP-BisSeq. (D) Same as C but only showing methylation levels derived from CTCF ChIP-BisSeq. In each case bound molecules show the same variation as the entire population. Only cytosines residing within the CTCF binding motif and with a minimal coverage of 10× are shown. In order to prevent over-plotting the points were jittered with a standard deviation of 2%.
(PDF)

Figure S3 5hmC marks LMRs in a cell-type specific fashion. (A) Replicate correlation for hMeDIP-seq. Shown is the log2 fold change of 5hmC between ES and NP in two biological replicates. (B) Correlation of hMeDIPseq and WG-BisSeq at LMRs during neuronal differentiation. Shown are the log2 fold change in 5hmC between ES and NP (y-axis) and change in DNA methylation percentage (x-axis). (C) Correlation of hMeDIP-seq and MeDIP-seq at LMRs during neuronal differentiation. Shown are the log2 fold change in 5hmC between ES and NP (y-axis) and change in DNA methylation percentage (x-axis).
(EPS)

Figure S4 5hmC enrichment at CTCF sites 5hmC enrichment at CTCF sites depends on CTCF binding. Shown are hMeDIP-seq enrichments in ES cells over bound and unbound CTCF motifs.
(EPS)

Table S1 Primer sequences used for qPCR.
(DOCX)
