# Supplementary figures and images for: Transcription Factor Occupancy Can Mediate Active Turnover of DNA Methylation at Regulatory Regions
Source: PLoS Genet. 2013 Dec 19;9(12):e1003994. doi: 10.1371/journal.pgen.1003994 (PMC3868540; doi:10.1371/journal.pgen.1003994)

**A**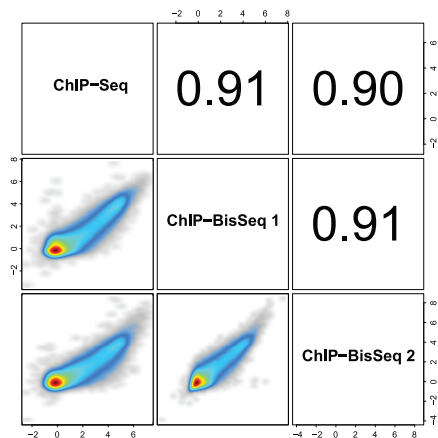**B**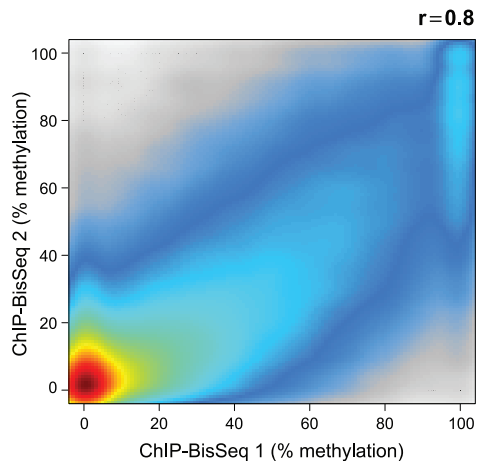**C**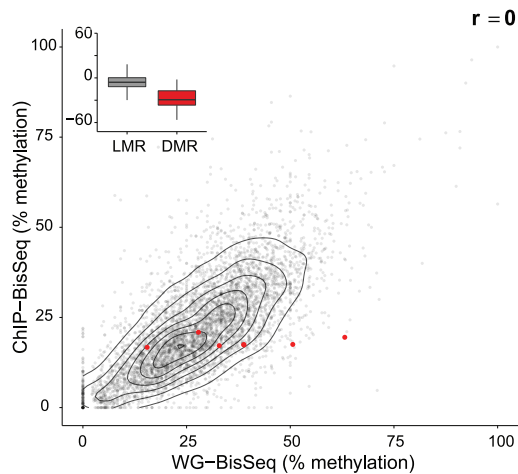**D**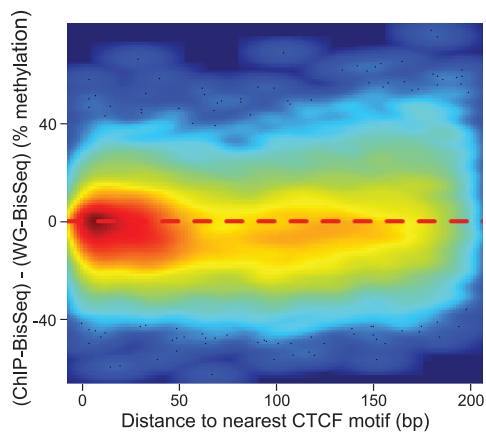

Supplement: Figure S1 — Genome-wide relation between transcription factor occupancy and methylation states. (A) Correlation of ChIP enrichments between CTCF ChIP-Seq (Stadler et al., Nature 2011) and the two CTCF ChIP-BisSeq replicates used in this study. (B) Correlation of methylation levels at individual CpGs between two CTCF ChIP-BisSeq replicates. Selected cytosines have a minimal coverage of 10 in both replicates. (C) Correlation of average methylation levels at regions 200 bp around all predicted CTCF sites between WG-BisSeq and a pool of both CTCF ChIP-BisSeq replicates. Selected regions have a minimal coverage of 10 in all cytosines used for the calculation of methylation levels in both WG-BisSeq and ChIP-BisSeq. (D) For individual cytosines within LMRs the methylation difference between ChIP-BisSeq and WG-BisSeq is correlated with the distance to the nearest CTCF motif center. (PDF) [file pgen.1003994.s001.pdf]

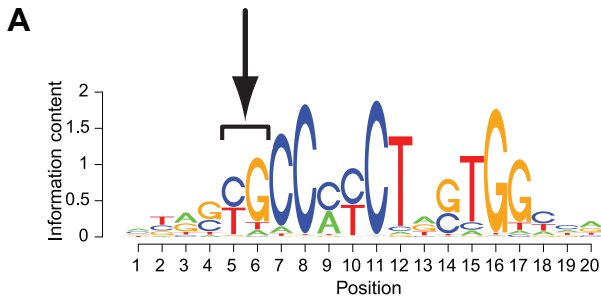

**B**

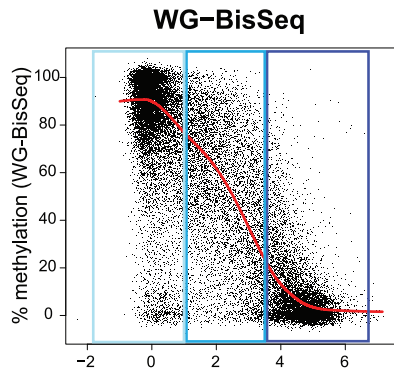

**C**

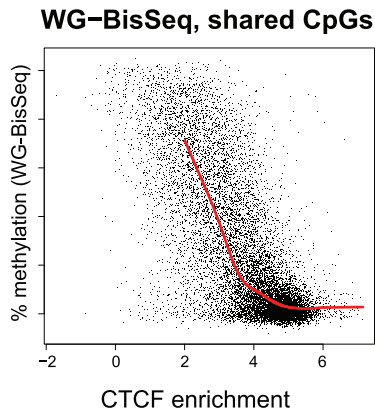

**D**

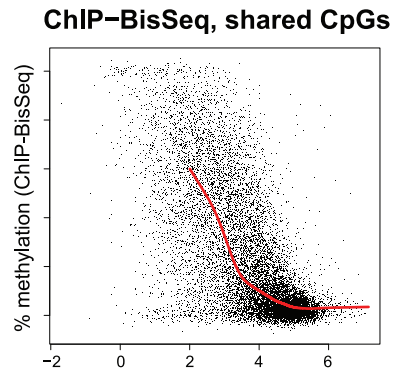

Supplement: Figure S2 — Relationship between binding strength and DNA methylation within the CTCF motif. (A) CTCF consensus motif used in this study. Here only cytosines are analyzed which are at position 5–6 of the motif. Out of all predicted sites containing a CpG within the motif (24.5% of all predicted sites) 42.2% have a CpG at this position. (B–D) Each point represents one individual CpG at position 5–6 of the PWM. (B) Correlation of methylation and CTCF enrichment identifies three classes of CTCF sites: unbound (light-blue), strongly bound and unmethylated (dark-blue), weakly bound with intermediate levels of methylation (blue). The red line represents a running mean measurement of methylation. (C) Same as B, but only showing cytosines covered in both WG-BisSeq and CTCF ChIP-BisSeq. (D) Same as C but only showing methylation levels derived from CTCF ChIP-BisSeq. In each case bound molecules show the same variation as the entire population. Only cytosines residing within the CTCF binding motif and with a minimal coverage of 10× are shown. In order to prevent over-plotting the points were jittered with a standard deviation of 2%. (PDF) [file pgen.1003994.s002.pdf]
