# Supplementary material for: Transcription Factor Occupancy Can Mediate Active Turnover of DNA Methylation at Regulatory Regions
Source: PLoS Genet. 2013 Dec 19;9(12):e1003994. doi: 10.1371/journal.pgen.1003994 (PMC3868540; doi:10.1371/journal.pgen.1003994)
Supplement: Table S1 — Primer sequences used for qPCR. (DOCX) [file pgen.1003994.s005.docx]

| **Table S1. Primer Sequences used for qPCR** | |  |
| --- | --- | --- |
|  |  |  |
| Genomic region | Forward | Reverse |
| Camta | GCTTCAGGGCTACAGAGTGC | AGTCAGAGGCTACCCCTGGT |
| Ciita | GCAAGCTGGAGAAAAAGCAC | TAGGATGAAGCCTGGGTGTC |
| Crhr2 | CGTGGCATTTATCGAAGTCA | GTGGTCAGGAGCTCTCCAAG |
| Zfp423 | CATTTGCTTCTCCGCAGATT | CATGTTTATGTCCGCTGCTG |
| Muc3 | CGGGTAGGAGACATCTCTGG | CCAGAGAGATGATGCTGGAAG |
| St6ga | CTCTTCTCGGTCACCCATTC | AATCACCCGCTGTGAATCAT |
| A6300Rik | CCCACGTCTCCATGGTTAAT | TTCTGTGCGTGGCTAAACAG |
| Zmynd8 | GGCGTTTCCTTGATTGACAT | AAGACAGGACCTGGAGGAGA |
| Interg LMR | CTTTGGCACACTGCCATCTA | CCTTTTCATGAGACCCGAAA |
| Interg3 | ATGCCCCTCAGCTATCACAC | GGACAGACATCTGCCAAGGT |
| Hprt | CCAAGACGACCGCATGAGAG | CAACGGAGTGATTGCGCATT |
| Gapdh | CTCTGCTCCTCCCTGTTCC | TCCCTAGACCCGTACAGTGC |
